# Population scale data reveals the antidepressant effects of ketamine and other therapeutics approved for non-psychiatric indications

**DOI:** 10.1038/s41598-017-01590-x

**Published:** 2017-05-03

**Authors:** Isaac V. Cohen, Tigran Makunts, Rabia Atayee, Ruben Abagyan

**Affiliations:** School of Pharmacy and Pharmaceutical Sciences, University of California San Diego, La Jolla, CA 92093 USA

## Abstract

Current therapeutic approaches to depression fail for millions of patients due to lag in clinical response and non-adherence. Here we provide new support for the antidepressant effect of an anesthetic drug, ketamine, by Inverse-Frequency Analysis of eight million reports from the FDA Adverse Effect Reporting System. The results of the examination of population scale data revealed that patients who received ketamine had significantly lower frequency of reports of depression than patients who took any other combination of drugs for pain. The analysis also revealed that patients who took ketamine had significantly lower frequency of reports of pain and opioid induced side effects, implying ketamine’s potential to act as a beneficial adjunct agent in pain management pharmacotherapy. Further, the Inverse-Frequency Analysis methodology provides robust statistical support for the antidepressant action of other currently approved therapeutics including diclofenac and minocycline.

## Introduction

The World Health Organization estimates depression as the 4^th^ highest disease burden in the world^1^. In majority of the countries lifetime depression prevalence ranges 8–12%^2–4^. Current standard of practice of depression treatment consists of five main classes of antidepressants, serotonin reuptake inhibitors (SSRIs) being the most common. Nearly half of psychiatric and primary care patients discontinue their antidepressant therapy prematurely^5^. The main reasons for the discontinuation of therapy include late onset of beneficial outcomes, lack of efficacy for a fraction of patients, adverse reactions, fear of drug dependence, and lack of mechanisms to enforce adherence^5^. The initial therapeutic effect of antidepressants is delayed by 2–3 weeks after the first dose and the optimal effect is delayed by 6–10 weeks^6^. The long lag period renders the standard of care antidepressants ineffective for suicidal patients who can’t afford to wait 2–6 weeks. Aside from the lag in antidepressant effects, there is insufficient evidence that antidepressants prevent suicide during long-term treatment^7^, and in many cases the antidepressant increases the risk of suicidal thoughts and actions^8^. Efficacy is another issue affecting depression treatment. In the STAR*D protocol study depression remission is 67% after every drug class and drug class combination is tried^9^.

Because of these problems, some clinicians have been driven to utilize other drugs, such as ketamine, for treatment resistant depression (TRD) patients^10–12^. Ketamine is a drug used illicitly as a hallucinogen and clinically as an anesthetic since 1970’s. It is given intravenously, almost exclusively, due to a lack of an approved oral formulation. There have been some clinical trials where ketamine shows acute efficacy in treating TRD^10,11^, bipolar depression^12^ and major depressive disorder with suicidal ideation^13^, but the number of subjects in these trials ranges from 20 to 57 patients. There are financial and ethical obstacles for a larger scale clinical trial. Here we sought larger scale statistical evidence of ketamine antidepressant action in the FDA Adverse Event Reporting System (FAERS) postmarketing database containing over eight million patient records. Although FAERS was originally intended to track frequent adverse events, with sufficient amount of data, it can also be used to track the beneficial outcomes indirectly through monitoring reductions of related complaint frequencies. Here we apply Inverse-Frequency Analysis (IFA), which looks for statistically significant values of the negative log odds ratio (LogOR).

**Figure 1:**
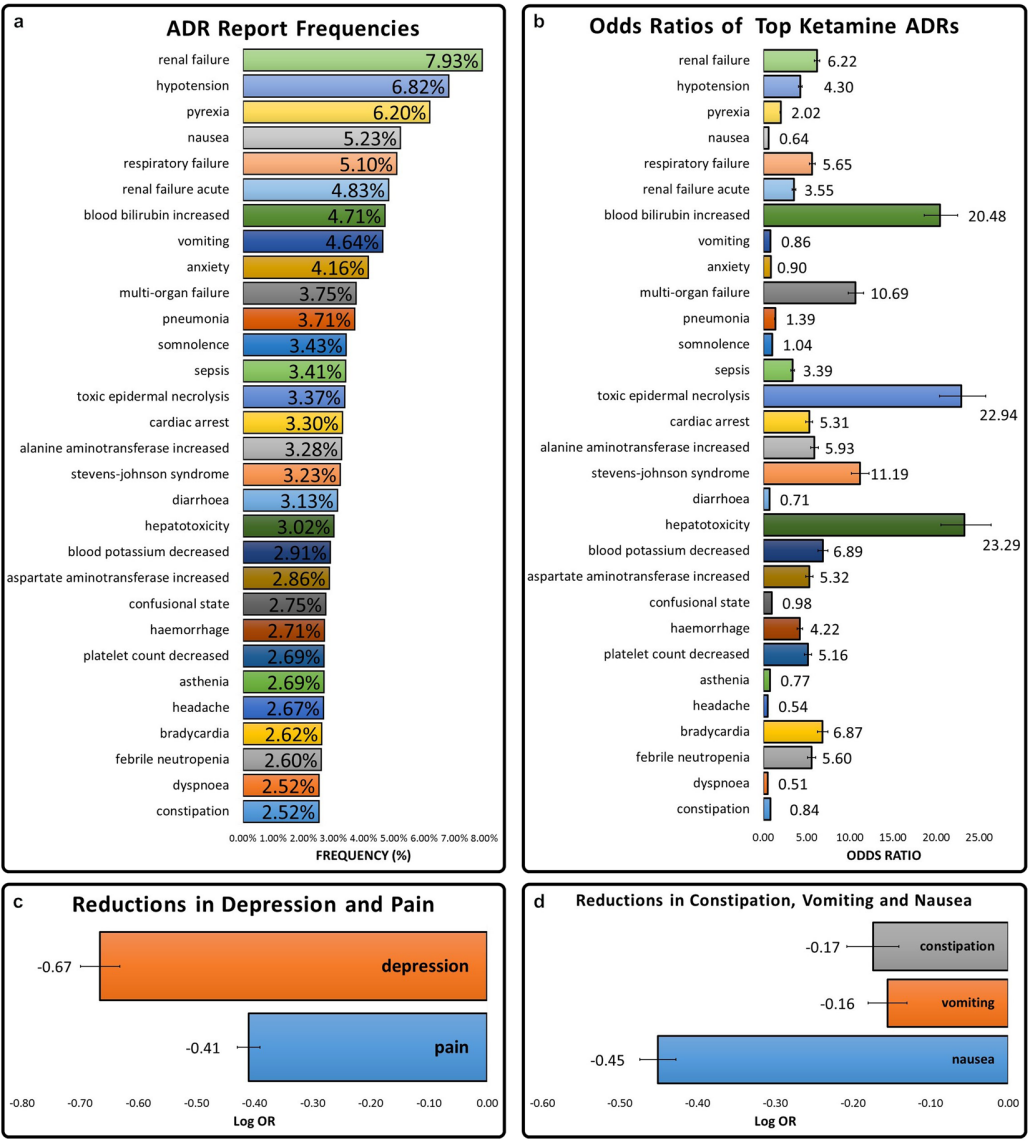
Legend: (**a**) Frequencies of adverse events in patients on FAERS who took ketamine. Adverse events above 2.5% were reported. (**b**) Odds ratios were calculated comparing adverse event rates of ketamine patients (n = 41,337) and pain patients (n = 238,516). (**c**) LogOR of pain and depression event rates were calculated from the ketamine and pain patient cohorts. Negative values showing protective effect of ketamine. (**d**) LogOR of constipation, vomiting, and nausea were calculated from the ketamine and pain patient cohorts. Negative values showing protective effect of ketamine.

We found that patients listed in the FAERS database who received ketamine in addition to other therapeutics had significantly lower frequency of reports of depression than patients who took any other combination of drugs for pain (LogOR −0.67 ± 0.034) (Fig. 1c). This reduction in depression is specific to ketamine and is known to be much more rapid than current antidepressants, making this observed effect very promising for treatment of patients with acute depressive or suicidal episodes^11^. These patients cannot afford to wait up to six weeks for reductions in their depressive symptoms. Pain reports were also significantly lower for ketamine patients (LogOR −0.41 ± 0.019) (Fig. 1c).

**Figure 2:**
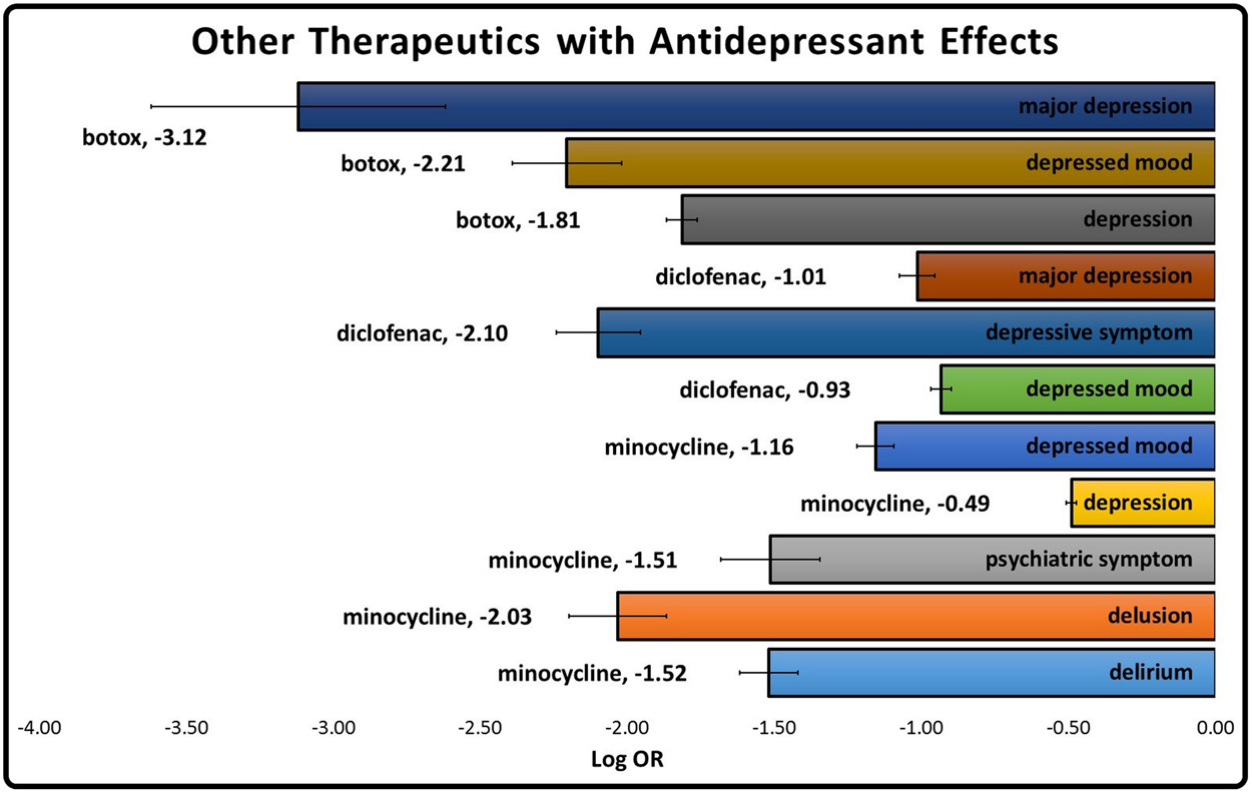
LogOR of psychiatric events were calculated from FAERS patients who used botox, diclofenac or minocycline. FAERS patients who took any drugs for the indication of depression were used as the control cohort. Negative values showing protective effect.

The analysis of the whole FAERS database revealed several other unintentional depression reducing drugs among antibiotics, cosmeceuticals and NSAIDS (Fig. 2). Our data supported previous studies that observed the psychiatric polypharmacology of *minocycline*, a tetracycline antibiotic^14^ (Fig. 2). The NSAID, *diclofenac*, was also observed to have some antidepressant properties (Fig. 2). It is theorized that both of these drugs may accomplish antidepressant effects through an anti-inflammatory mechanism^15^. Because of the antidepressant activity of several NSAIDs, we further separated the non-ketamine pain cohort. Ketamine patients were then compared to patients who received any other combination of drugs for pain *excluding* NSAIDs. It was observed that depression event rates remained low (LogOR −0.56 ± 0.035) (Fig. 2).

The reduction of depression rates in ketamine patient records makes a case for study of ketamine as a psychiatric drug. These results imply that ketamine may be further explored as a monotherapy or adjunct therapy for depression. It should also be noted that FAERS data revealed that ketamine use lead to renal side effects and awareness and caution in patients with renal or hepatic impairment may be warranted (Fig. 1a and b).

As an important side note, we also evaluated efficacy and side effects with the use of ketamine for *pain management*. We found that patients who were on ketamine had reduced opioid induced side effects including constipation (LogOR −0.17 ± 0.023), vomiting (LogOR −0.16 ± 0.025), and nausea (LogOR −0.45 ± 0.034) than patients who received any other combination of drugs for pain indications (Fig. 1d). Our data supports ketamine’s opioid-sparing properties and alludes to the fact that patients may receive benefits of improved pain, reduced requirement of opioids, and ultimately less opioid reduced side effects.

The results of this study support previous small scale studies’ conclusions that ketamine is a good monotherapy or adjunct therapy for depression. In clinical practice ketamine would be especially useful for depression because of the quick onset of its action compared to existing first line therapies^10–13^. Regardless of the causative mechanism ketamine appears to have therapeutic potential for TRD. Further, the potential to reduce many of the most complained side effects of opioid treatment makes ketamine adjunct therapy for pain seem desirable.

Overall, this study demonstrates that the therapeutic potential of ketamine can be derived from appropriate statistical analysis of existing population scale data. This study also outlines a methodology for discovering off label pharmacology of existing approved drugs. This method can be applied to other indications and may reveal new important uses of already approved drugs, providing reliable justification for new indications without large investments in additional clinical trials.

## Methods

This study used data from FDA Adverse Effect Reporting System database (FAERS)^16^ and its legacy version, Adverse Effect Reporting System database (AERS)^17^, to perform a retrospective data analysis on the drugs of interest. Data from FAERS and AERS is available online at: http://www.fda.gov/Drugs/GuidanceComplianceRegulatoryInformation/Surveillance/AdverseDrugEffects/ucm082193.htm.

### FDA Adverse Event Reporting System

The FAERS database was created to support FDA’s post marketing surveillance on drugs and biologic therapeutics. It contains adverse reaction and medication error reports sent to the FDA through MedWatch, the FDA Safety Information and Adverse Event Reporting Program. Reporting is voluntary and is done by patients, family members, legal representatives, doctors, pharmacists and other healthcare providers. If any party reports an adverse effect to the manufacturer, the manufacturer is legally obligated to forward the report to the FDA^18^. Data is available online in quarterly format for AERS^17^ from the first quarter of 2004 to the third quarter of 2012 and for FAERS^16^ from the fourth quarter of 2012 to the first quarter of 2016.

### Combining and normalizing the data set

For our study AERS and FAERS data sets were homogenized by modifying original text tables to produce a consistent table field structure. The combining was performed by individually downloading the FAERS and AERS quarterly reports in dollar separated text format (*.TXT). The names of columns were also homogenized and the columns missing from older releases were added with empty values.

The study used over 8 million adverse event reports from first quarter of 2004 to the first quarter of 2016. All the quarterly files from 2004 to 2016 were combined into a master file which was used as the primary source for analysis. The drug names and indications were submitted without controlled vocabulary. All synonyms, capitalizations, and misspellings were recognized and assigned to a single value. The same homogenization of terms was performed for other drugs of interest, adverse reactions, and indications.

### Data limitations

Due to the absence of a full set of medical records and the voluntary nature of the FAERS and AERS databases, the data in the study represents a subset of actual cases. Therefore, the frequencies and odds ratios of adverse events computed were *not* true absolute population frequencies. However, the combined set of records had provided sufficient statistical power for the analysis. Out of 8 million reports, 279,853 reports were used for analysis of ketamine in Fig. 1. Two cohorts for ketamine (K) patients and pain (P) patients with 41,337 and 238,516 patients respectively.

### Statistical analysis

#### Descriptive Statistics

The top side effects with frequencies above 2.5% were reported for Fig. 1a and b. Frequency of each side effect was calculated by the equation:

1 $$({Number}\,{of}\,{Adverse}\,{Effect}\,{Events})/({Number}\,{of}\,{Patient}\,{Records})={Frequency}$$

#### Comparative Statistics

Patient cohorts adverse event report rates were compared via the Odds Ratio (OR) and the Log Odds Ratio (LogOR). The OR was calculated by the following equation:

2 $$\begin{array}{rcl}{OR} & = & (a/b)/(c/d)\end{array}$$

a = Number in exposed group with adverse eventb = Number in control group with adverse eventc = Number in exposed group with no adverse eventd = Number in control group with no adverse eventStandard Error (SE) was calculated by the following equation:

3 $$S{E}_{OR}={square} \mbox{-} {root}(1/a+1/b+1/c+1/d)$$

*variables a, b, c, and d as defined in Eq. 2.

The 95% Confidence Interval was used to compute error bars for Fig. 1b and was computed using the following formula:

4 $${95} \% \,CI=exp\{OR-1.96\times S{E}_{OR}\}\,to\,exp\{OR+1.96\times S{E}_{OR}\}$$

*variables OR and SE_OR_ as defined in Eqs 2 and 3.

The LogOR was calculated for Figs 1c,d and 2 by the following equation

5 $$Log\,OR=Log(OR)$$

*variable OR as defined in Eq. 2.

The standard error for the LogOR was calculated by the following equation:

6 $$S{E}_{LogOR}={square} \mbox{-} {root}(1/a+1/b+1/c+1/d)$$

*variables a, b, c, and d as defined in Eq. 2.

LogOR Error was used to compute error bars for Figs 1c,d and 2 and was computed using the following formula:

7 $$LogOR\,Error=\pm 1.96\times S{E}_{LogOR}$$

*variable SE_LogOR_ as defined in Eq. 6.
