# Publisher Correction: Population scale data reveals the antidepressant effects of ketamine and other therapeutics approved for non-psychiatric indications

**DOI:** 10.1038/s41598-017-14992-8

**Published:** 2017-12-05

**Authors:** Isaac V. Cohen, Tigran Makunts, Rabia Atayee, Ruben Abagyan

**Affiliations:** School of Pharmacy and Pharmaceutical Sciences, University of California San Diego, La Jolla, CA 92093 USA

*Scientific Reports* **7**:1450; doi:10.1038/s41598-017-01590-x; Article published online 03 May 2017

This Article contains errors in the legend of Figure 1.

“(b) Odds ratios were calculated comparing adverse event rates of ketamine patients (n = 41,337) and pain patients (n = 238,516)”.

should read:

“(b) Odds ratios were calculated comparing adverse event rates of ketamine records (n = 41,337) and pain records (n = 238,516)”.

In addition, in the Methods section under the subheading ‘Data limitations’:

“Out of 8 million reports, 279,853 reports were used for analysis of ketamine in Fig. 1. Two cohorts for ketamine (K) patients and pain (P) patients with 41,337 and 238,516 patients respectively”.

should read:

“Out of 8 million reports, 279,853 records were used for analysis of ketamine in Fig. 1. Two cohorts for ketamine (K) patients and pain (P) patients with 41,337 and 238,516 records respectively”.

